# Selective activation of cellular stress response pathways by fumaric acid esters

**DOI:** 10.1002/2211-5463.13833

**Published:** 2024-05-24

**Authors:** Katrin Erler, Niklas Krafczyk, Holger Steinbrenner, Lars‐Oliver Klotz

**Affiliations:** ^1^ Nutrigenomics Section, Institute of Nutritional Sciences Friedrich Schiller University Jena Germany

**Keywords:** AKT, alkylation, FOXO, glutathione, insulin signaling, NRF2

## Abstract

The cellular response to oxidants or xenobiotics comprises two key pathways, resulting in modulation of NRF2 and FOXO transcription factors, respectively. Both mount a cytoprotective response, and their activation relies on crucial protein thiol moieties. Using fumaric acid esters (FAEs), known thiol‐reactive compounds, we tested for activation of NRF2 and FOXO pathways in cultured human hepatoma cells by dimethyl/diethyl as well as monomethyl/monoethyl fumarate. Whereas only the diesters caused acute glutathione depletion and activation of the stress kinase p38^MAPK^, all four FAEs stimulated NRF2 stabilization and upregulation of NRF2 target genes. However, no significant FAE‐induced activation of FOXO‐dependent target gene expression was observed. Therefore, while both NRF2 and FOXO pathways are responsive to oxidants and xenobiotics, FAEs selectively activate NRF2 signaling.

## Graphical abstract

NRF2 and FOXO transcription factors respond to oxidants and xenobiotics. We analyzed the activation of NRF2 and FOXO by fumaric acid esters in HepG2 cells. Whereas only the diesters caused acute glutathione depletion and activation of the stress kinase p38^MAPK^, all tested compounds stimulated NRF2 signaling. No FOXO‐dependent response was observed, indicating that FAEs selectively activate NRF2 signaling.

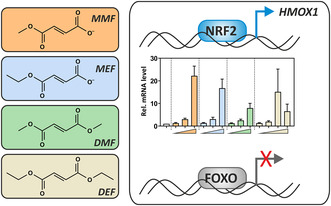

**Abbreviations**
DEF: diethylfumarate
DEM: diethylmaleate
DMF: dimethylfumarate
FAE: fumaric acid ester
FOXO: Forkhead box, class O, protein
G6PC: glucose‐6‐phosphatase, catalytic subunit
G6PD: glucose‐6‐phosphate dehydrogenase
GAPDH: glyceraldehyde‐3‐phosphate dehydrogenase
GSH: glutathione
GSSG: glutathione disulfide
HCAR: hydroxy‐carboxylic acid receptor
HMOX1: heme oxygenase 1
KEAP1: Kelch‐like ECH‐associated protein 1
MAPK: mitogen‐activated protein kinase
MEF: monoethylfumarate
MMF: monomethylfumarate
NQO1: NAD(P)H:quinone oxidoreductase 1
NRF2: NFE2 related/like bZIP transcription factor 2, NFE2L2
ROS: reactive oxygen species
SELENOP: selenoprotein P

Cellular responses to stressful stimuli, including reactive oxygen species (ROS) and xenobiotics, frequently overlap, with both oxidative stress and xenobiotics eliciting acute and delayed cellular adaptations, the latter usually being at the level of transcriptional regulation. There are, however, also a few intracellular molecules and pathways modulated in parallel by both oxidative stress and xenobiotics. Among the shared cytoprotective pathways are those that lead to activation of NRF2 (NFE2‐like bZIP transcription factor 2, NFE2L2) and FOXO (forkhead box, class O) transcription factors [1]. At a molecular level, crucial thiol moieties contribute to these processes, as they may not only be oxidized but also form adducts with electrophilic xenobiotics. In the case of NRF2 signaling, cysteine thiols of kelch‐like ECH associated protein 1 (KEAP1) render it a target protein that controls the activity of NRF2 by mediating its proteasomal degradation under non‐stressed conditions. Oxidation or alkylation of KEAP1 interrupts this regulatory circuit, resulting in stabilization and nuclear translocation of NRF2 as well as induction of NRF2 target genes, including genes encoding ROS‐detoxifying (antioxidant) enzymes as well as enzymes required for biosynthesis of the reducing equivalents glutathione (γGlu‐Cys‐Gly, GSH) and nicotinamide adenine dinucleotide phosphate (NADPH) [2, 3]. Similar to the KEAP1/NRF2 system, transcription factors of the FOXO family (as well as proteins upstream in the cascade, modulating FOXO activity) contain cysteine residues that are involved in their redox regulation [4, 5]. FOXO signaling, accordingly, is affected by xenobiotics and oxidants [6, 7, 8]. FOXOs, in turn, control the expression of genes encoding several antioxidant enzymes [9].

There are reports indicating that the redox‐sensitive NRF2‐ and FOXO transcriptional regulatory systems may be connected, for example through transcriptional regulation of antioxidant enzymes by FOXOs and the consecutive modulation of steady‐state levels of ROS such as hydrogen peroxide, which, in turn, affects the activity of KEAP1/NRF2 (for review, see ref. [1]). Moreover, several flavonoids were shown to stimulate both NRF2‐ and FOXO‐dependent signaling in cultured human cells [10]. In this regard, exposure of the model organism *Caenorhabditis elegans* (*C. elegans*) to the electrophilic and adduct‐forming compound diethylmaleate (DEM) caused glutathione depletion and, at lower levels of exposure, a significant lifespan extension. Both the FOXO and the NRF2 orthologs in the worms (DAF‐16 and SKN‐1, respectively) were involved in the DEM‐mediated increase in lifespan [11].

The course of events and the mode of the potential collaboration between these two redox‐sensitive transcriptional regulatory pathways are ill‐defined. For example, if both pathways react to thiol‐modulating agents and to oxidative stress, does one of the pathways predominate the cellular response, or do both collaborate? Can one pathway be stimulated independently of the other?

Here, we begin to address these questions by making use of fumaric acid esters (FAEs) as a group of electrophilic substances with different membrane permeation properties: the uncharged diesters, diethyl fumarate (DEF) and dimethyl fumarate (DMF), as compared to the monoesters, monoethyl fumarate (MEF) and monomethyl fumarate (MEF), that are ionized at physiological pH (Fig. 1A). FAEs were chosen as compounds to analyze activation of both NRF2 and FOXO pathways for two reasons. (a) Esters of both fumaric acid as well as maleic acid (including the above‐mentioned DEM) were found to stimulate the expression of genes encoding GSH S‐transferases (GSTs) and NAD(P)H:quinone oxidoreductase 1 (NQO1), now known as genes regulated by NRF2, already in 1990 [12]. (b) FAEs are being employed pharmacologically and are, therefore, of rather general interest: DMF is approved for the treatment of a form of multiple sclerosis (MS), relapsing–remitting MS [13, 14]. It is also used in the treatment of psoriasis, another inflammatory disease, often in combination with other FAEs, such as MEF [14, 15, 16, 17]. The exact mechanism of action has not been resolved entirely, but both immunomodulatory and antioxidant effects (through stimulation of signaling cascades) were proposed to contribute to the efficacy of FAEs.

**Fig. 1:**
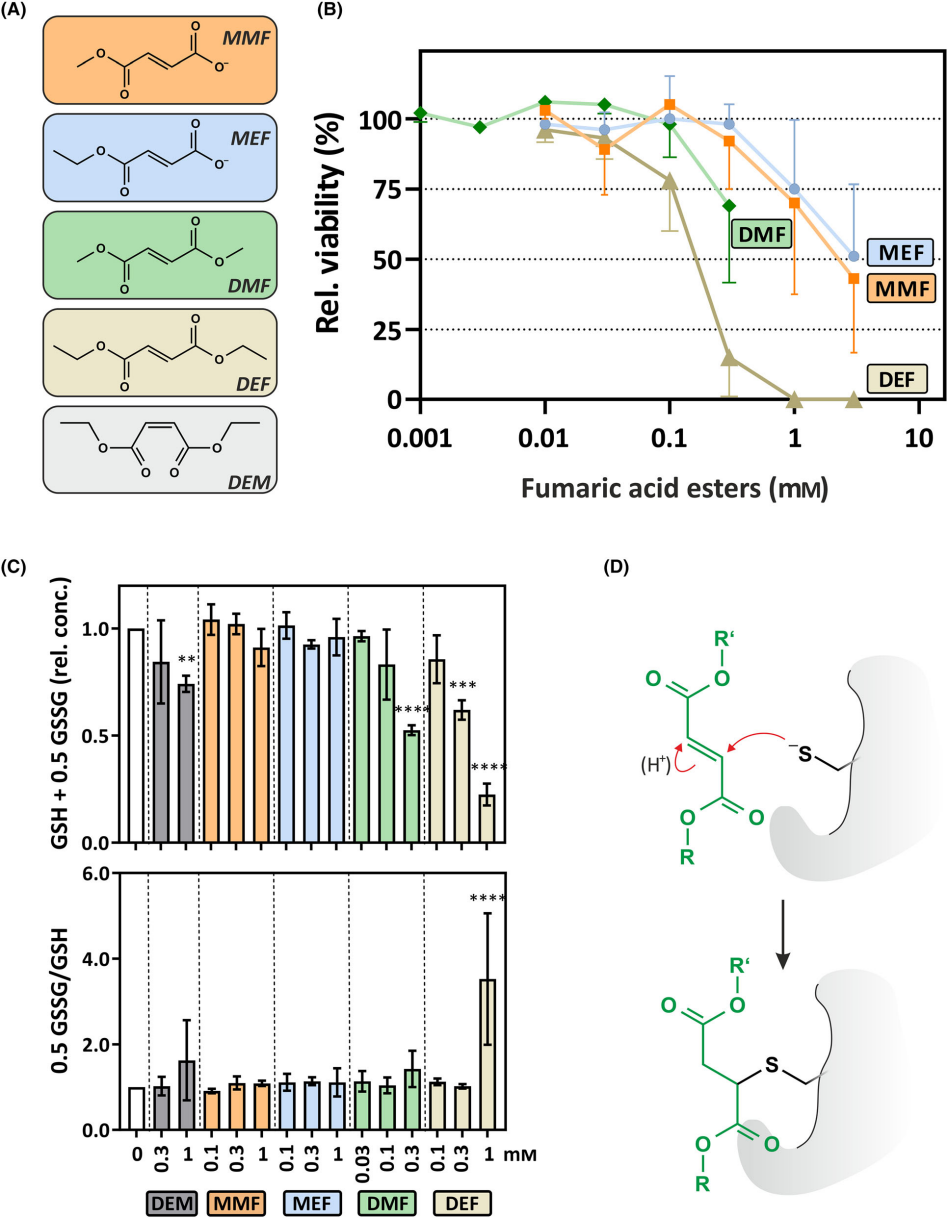
Effects of fumaric acid esters (FAEs) on viability and glutathione levels in HepG2 cells. (A) Structures of FAEs and of diethylmaleate (DEM): monomethylfumarate (MMF), monoethylfumarate (MEF), dimethylfumarate (DMF), diethylfumarate (DEF). (B) Dose‐dependent cytotoxicity of FAEs. HepG2 cells were exposed to FAEs at the indicated concentrations or to DMSO (solvent control) in serum‐free medium for 24 h. Cell viability was assessed using a neutral red assay. Relative values were calculated by setting the viability of cells exposed to solvent control to 100%. Data are given as means of four independent experiments +SD/−SD. (C) Glutathione (0.5 GSSG + GSH) levels and redox ratio (0.5 GSSG/GSH) in HepG2 cells exposed to FAEs, DEM (as positive control) or DMSO (“0 mm”; solvent control) at the indicated concentrations for 60 min in serum‐free medium. Data are means of three independent experiments ± SD. Data were normalized against control conditions (DMSO; “0 mm”), which were set to 1. Under these conditions, glutathione and glutathione disulfide concentrations were as follows (mean ± SD): [GSH] = 55.1 ± 3.6 nmol·mg^−1^ protein; [GSSG] = 0.4 ± 0.1 nmol·mg^−1^ protein. Statistically significant differences were determined by one‐way ANOVA with Dunnett's *post‐hoc* test. **P < 0.01, ***P < 0.001, ****P < 0.0001. (D) Scheme depicting a Michael‐type addition of a FAE to a reactive protein cysteinyl residue, resulting in protein succination.

Cultured HepG2 human hepatoma cells were chosen as a model to investigate the effects of FAEs as they (a) have been characterized previously in our laboratory regarding their harboring both NRF2 and FOXO signaling cascades [18, 19] and (b) physiologically, liver—although not the ultimate target tissue of FAEs—would be expected to be exposed to systemically applied FAEs via portal blood *in vivo*. (c) Moreover, psoriasis has been found to be associated with an increased risk to develop liver injuries such as non‐alcoholic fatty liver disease (NAFLD), and systemic use of FAEs may elicit both hepatoprotective and hepatotoxic effects [20].

We found that all of the tested FAEs, independent of their acute thiol‐depleting effects, stabilized NRF2 and upregulated the expression of NRF2 target genes. In contrast, despite, in part, affecting FOXO cytoplasmic/nuclear shuttling, FAEs did not affect the expression of FOXO target genes. In summary, while both NRF2 and FOXO pathways are generally responsive to oxidants and xenobiotics, FAEs selectively activate NRF2 signaling.

## Experimental procedures

### Materials

Chemicals were purchased from Merck/Sigma (Darmstadt, Germany) or Carl Roth GmbH (Karlsruhe, Germany), unless noted otherwise. “μ‐Slides VI 0.4” used for analyses of subcellular localization of EGFP‐FOXO1 were obtained from ibidi (Martinsried, Germany). FAEs and DEM were kept as stock solutions in DMSO, with the following stock concentrations: MMF (Merck, Cat# 651419; 130.1 g·mol^−1^) and MEF (Merck, Cat# 128422; 144.13 g·mol^−1^) at 3 m, DEF (Merck, Cat# D95654; 172.18 g·mol^−1^) and DEM (Merck, Cat# D97703; 172.18 g·mol^−1^) at 6 m, DMF (Merck, Cat# 242926; 144.13 g·mol^−1^), owing to relatively poor solubility, at 0.3 m.

### Cultivation of HepG2 cells

HepG2 human hepatoma cells were obtained from the German Collection of Microorganisms and Cell Cultures (DSMZ, Braunschweig, Germany, Cat# ACC 180) and held in Dulbecco's modified Eagle's medium (DMEM, low glucose, GlutaMAX supplement; Thermo Fisher Scientific, Waltham, MA, USA, Cat# 21885108), supplemented with 10% (v/v) fetal bovine serum (Merck/Sigma, Cat# S0615), 100 U·mL^−1^ penicillin, 100 μg·mL^−1^ streptomycin (Merck/Sigma, Cat# P4333) and 1% (v/v) non‐essential amino acids (in MEM; Merck/Sigma, Cat# M7145), at 37 °C in a humidified atmosphere with 5% (v/v) CO_2_. Passages 14 to 27 (counted from the first passage of the batch received from DSMZ in our laboratory) were used for the experiments described here. Cell viability was assessed using Neutral Red solution (Merck/Sigma, Cat# N2889) according to standard procedures.

### Determination of intracellular glutathione concentrations

Glutathione and glutathione disulfide concentrations were determined by HPLC following derivatization of thiols in cell lysates with orthophthaldialdehyde (OPA) as described [21], with minor modifications. In brief, cells were collected in 0.01 N HCl, followed by sonication on ice and precipitation of proteins by the addition of 2 N perchloric acid to a final concentration of 0.67 N. Following neutralization of the supernatant using 0.5 mm sodium phosphate buffer (pH 7.0), thiols were derivatized with OPA (final concentration: 0.065 m) at room temperature in the dark for 60 min. OPA adducts of GSH were analyzed using HPLC on a Nucleodur C18 Pyramid 5 μ RP column (4 × 250 mm; Macherey‐Nagel, Düren, Germany) and gradient elution with (A) 98% of 50 mm sodium acetate (pH 7.0)/2% acetonitrile and (B) 80% acetonitrile/20% 50 mm sodium acetate (pH 7.0) as eluents. Detection of adducts was by fluorescence (Ex: 350/Em: 420 nm).

For analysis of GSSG content, all GSH was alkylated by the addition of N‐ethylmaleimide (NEM, final conc. 9.1 mm) to the deproteinized supernatants. Excess NEM was removed by addition of N‐acetyl cysteine (final conc. 8.3 mm). Subsequently, GSSG was reduced to GSH using dithiothreitol (DTT; final conc. 7.7 mm), followed by detection of GSH by OPA‐derivatization as above.

### Analysis of FOXO1 subcellular localization

HepG2 cells were grown for 28–30 h to approx. 60% confluence on 6‐channel‐microscopy slides with a coverslip bottom (“μ‐Slides VI 0.4”; ibidi, Cat# 80606). Cells were transiently transfected with 2 μg of plasmid DNA encoding an EGFP‐coupled form of FOXO1 [21] and 6 μL GenJet transfection reagent (tebu‐bio, Offenbach, Germany) for 16–18 h. Following a preincubation with serum‐free medium for 4–5 h, cells were exposed to bovine insulin (100 nm; Merck/Sigma, Cat# I0516) and/or DMF, DEF (or the respective solvent controls) while being observed under the microscope. The microscopy slides were then placed in an incubator box (Okolab, Ottaviano, Italy), held at 37 °C/5% CO_2_ and analyzed. Fluorescence microscopic images were taken at multiple time points (Nikon Eclipse Ti fluorescence microscope). Transfected cells (as judged by their green fluorescence) were grouped into three categories with respect to the predominant subcellular localization of the EGFP signal (“cytoplasmic”, “cytoplasmic/nuclear” or “nuclear”).

### Quantitative RT‐PCR (qRT‐PCR)

HepG2 cells were seeded in 6‐well plates and were starved for 18 h in DMEM supplemented with 100 U·mL^−1^ penicillin, 100 μg·mL^−1^ streptomycin and 1% (v/v) non‐essential amino acids. Afterwards, they were exposed to different concentrations of FAEs (MMF, MEF, DMF, DEF) or DEM and/or 100 nm insulin or 0.1% (v/v) DMSO as solvent control diluted in serum‐free DMEM. Exposure was for 4 or 16 h, respectively, followed by lysis of cells and isolation of total RNA using the RNeasy Mini kit (Qiagen, Hilden, Germany) according to the manufacturer's instructions.

1 μg of total RNA was converted to cDNA using RevertAid reverse transcriptase (Thermo Scientific) according to the manufacturer's instructions, and subjected to qPCR analysis on a CFX Connect cycler (Bio‐Rad Laboratories AG, Munich, Germany) using SsoAdvanced Universal SYBR Green Supermix (Bio‐Rad). The housekeeping gene *HPRT1* transcript was used for normalization of mRNA levels. Sequences of primers used are listed in Table 1.

**Table 1:** Sequences of primers used for qPCR.

Target	Primer sequences (5′→3′)	GenBank entry
*G6PC*	TTCCCTGTAACCTGTGAGACTG AGATGGAAAGAGTAGATGTGACCAT	NM_000151.4
*G6PD*	GCAAACAGAGTGAGCCCTTC GGCCAG CCACATAGGAGTT	NM_001360016.2
*GCLC*	CATGGGATTTGGAATGGGCAA GAGATGCAGCACTCAAAGCC	NM_001498.4
*HMOX1*	CTGCTCAACATCCAGCTCTTTG CAGCATGCCTGCATTCACAT	NM_002133.3
*HPRT1*	GGGGACATAAAAGTAATTGGTGGAG CTGACCAAGGAAAGCAAAGTCTG	NM_000194.3
*NQO1*	GCTCACCGAGAGCCTAGTTC TCCTCTCTGAGTGAGCCAGT	NM_000903.3
*SELENOP*	AACTGCTCTCTCACGACTCTC AGCATTTGGTGCTCCTGGTT	NM_005410.4

### Western blotting

SDS/PAGE and western blotting were performed according to standard procedures [6], using polyvinylidene membranes for blotting and the following primary antibodies (all from Cell Signaling Technology, Danvers, MA, USA): anti‐phospho‐FOXO1 (Thr24)/FOXO3a (Thr32) (rabbit polyclonal, Cat# 9464), anti‐phospho‐AKT (S473) (rabbit monoclonal, D9E, Cat# 4060), anti‐phospho‐p38^MAPK^ (Thr180/Tyr182) (rabbit monoclonal, D3F9, Cat# 4511), anti‐FOXO1 (rabbit monoclonal, C29H4, Cat# 2880), anti‐AKT (rabbit monoclonal, C67E7, Cat# 4691), anti‐p38^MAPK^ (rabbit polyclonal, Cat# 9212), anti‐NRF2 (rabbit monoclonal, D1Z9C, Cat# 12721). Detection of glyceraldehyde 3‐phosphate dehydrogenase (GAPDH, mouse monoclonal antibody from Sigma, GAPDH‐71.1, Cat# G8795) as well as staining of membranes for detection of protein using Ponceau S (Merck/Sigma, Cat# P7170) served as loading controls. Incubation with secondary antibody [horseradish peroxidase (HRP)‐conjugated anti‐rabbit IgG (Cell Signaling Technology, Cat# 7074) or HRP‐coupled anti‐mouse IgG (Thermo Fisher Scientific, Cat# 31430)] was followed by detection using chemiluminescent HRP substrate (SuperSignal West Pico and Femto; Thermo Fisher Scientific). Images were acquired using a ChemiDoc MP analyzer and Image Lab software, version 6.0.1 (Bio‐Rad, Hercules, CA, USA). Normalization of signals for NRF2, phospho‐FOXO1, and phospho‐AKT was over total protein signal following staining of membranes using Ponceau S. The signals for phospho‐p38^MAPK^ were normalized over total p38^MAPK^.

### Statistical analysis

Means were calculated from at least three independent experiments, and error bars represent standard deviation (SD). Statistical analyses were performed using graphpad prism software, version 8 and above (GraphPad Software, San Diego, CA). Statistically significant differences were determined using a one‐way ANOVA with Dunnett's *post‐hoc* test. Values of *P* < 0.05 were considered statistically significant and denoted as follows: *P < 0.05, **P < 0.01, ***P < 0.001, ****P < 0.0001.

## Results

### Fumaric acid diesters, but not monoesters, elicit acute depletion of glutathione and activation of the stress kinase p38^MAPK^

**Fig. 2:**
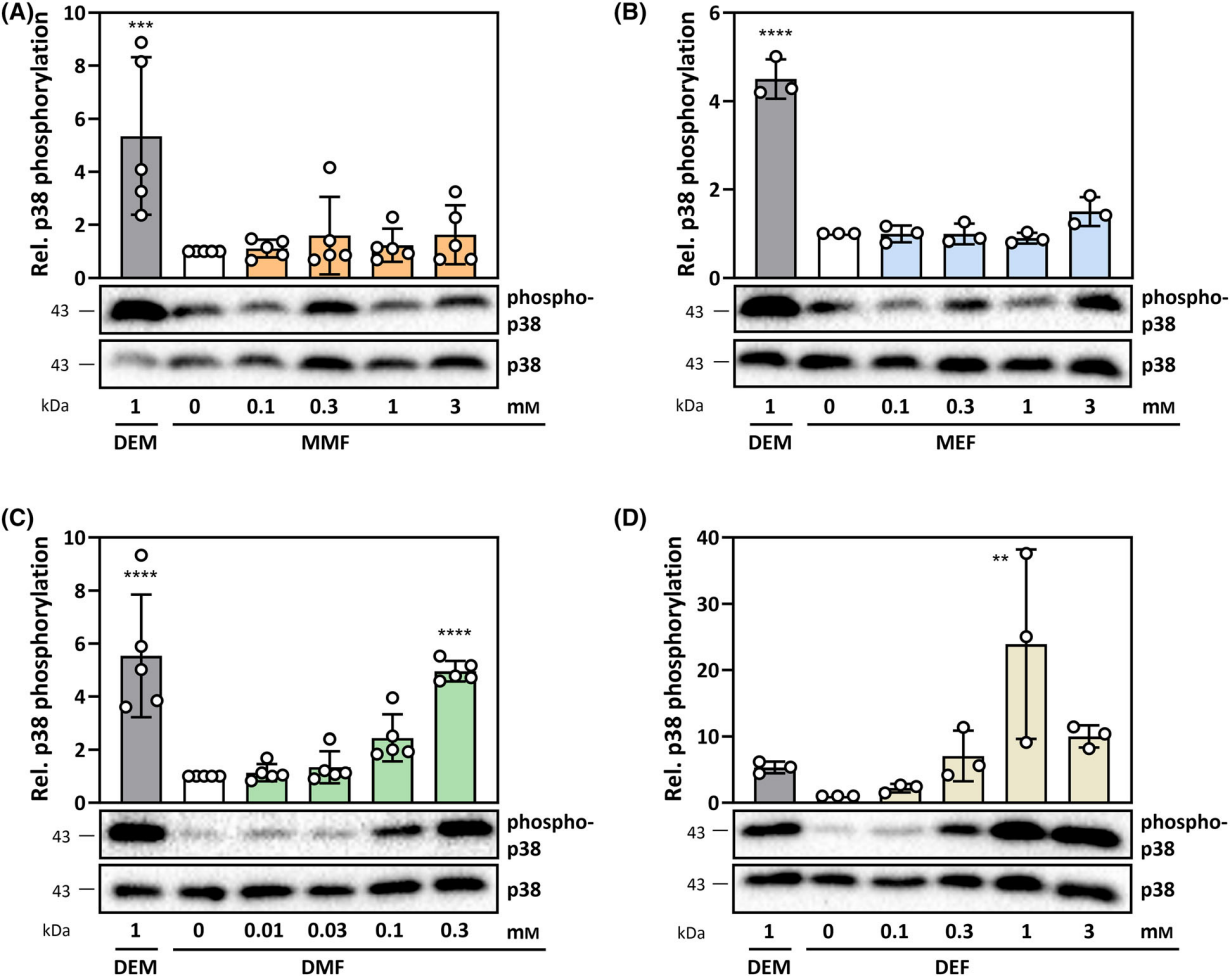
Phosphorylation of p38^MAPK^ in HepG2 cells exposed to FAEs for 60 min. (A) Monomethylfumarate, (B) Monoethylfumarate, (C) Dimethylfumarate, (D) Diethylfumarate. Diethylmaleate (DEM) was used as positive control in all experiments. Blots are representative of at least three independent experiments. Positions of protein markers closest to the detected band are indicated on the left (in kDa). Bar graphs represent densitometric analyses of the blots, means ± SD. Statistically significant differences from control (white bars) were determined by one‐way ANOVA with Dunnett's *post‐hoc* test. **P < 0.01, ***P < 0.001, ****P < 0.0001.

HepG2 cells were exposed to various concentrations of any of four FAEs (see Fig. 1A), mono‐ or dimethylfumarate (MMF, DMF) or mono‐ or diethylfumarate (MEF, DEF), followed by analysis of cell viability (Fig. 1B) and two parameters reflecting the capability of eliciting an acute stress response, depletion of glutathione (Fig. 1C), e.g. through a reaction depicted in Fig. 1D, and activation of p38^MAPK^ (Fig. 2). FAE cytotoxicity was assessed after a 24 h‐incubation period and was highest with the diesters, DEF and DMF (Fig. 1B), likely owing to their higher lipophilicity and better membrane permeation [22]. The highest final concentration achievable with DMF in our hands was 0.3 mm, which was less toxic than the same concentration of DEF, but more so than MMF or MEF (Fig. 1B).

Only DMF and DEF caused statistically significant glutathione depletion in exposed cells (Fig. 1C). Moreover, only DEF at 1 mm elicited oxidation of reduced glutathione (GSH) to form GSSG and result in a significantly elevated GSSG/GSH ratio, indicative of an impaired redox homeostasis (Fig. 1C); overall, this suggests that the glutathione depletion observed was largely due to alkylation. More rapid access to the intracellular milieu is likely to contribute to the higher acute glutathione depletion by the fumaric acid diesters as well as interaction with cellular protein thiols, resulting in a cellular stress response (Fig. 1D). This FAE reactivity pattern is also reflected at a cellular signaling level: only DMF and DEF elicited statistically significant phosphorylation (activation) of the kinase p38^MAPK^, which is known to respond to a variety of stress‐inducing stimuli (Fig. 2).

The *cis*‐isomer of DEF, DEM (Fig. 1A), was used as a positive control for several parameters in this study, based on previous work on its effects in HepG2 cells [18, 21]. Similar to the fumaric acid diesters, DEM induced rapid glutathione depletion and p38^MAPK^ phosphorylation **(**Figs 1C and 2 **)**.

### All FAEs stimulate NRF2 stabilization and signaling but differ with respect to active concentrations and the time courses of upregulation of NRF2 target genes

**Fig. 3:**
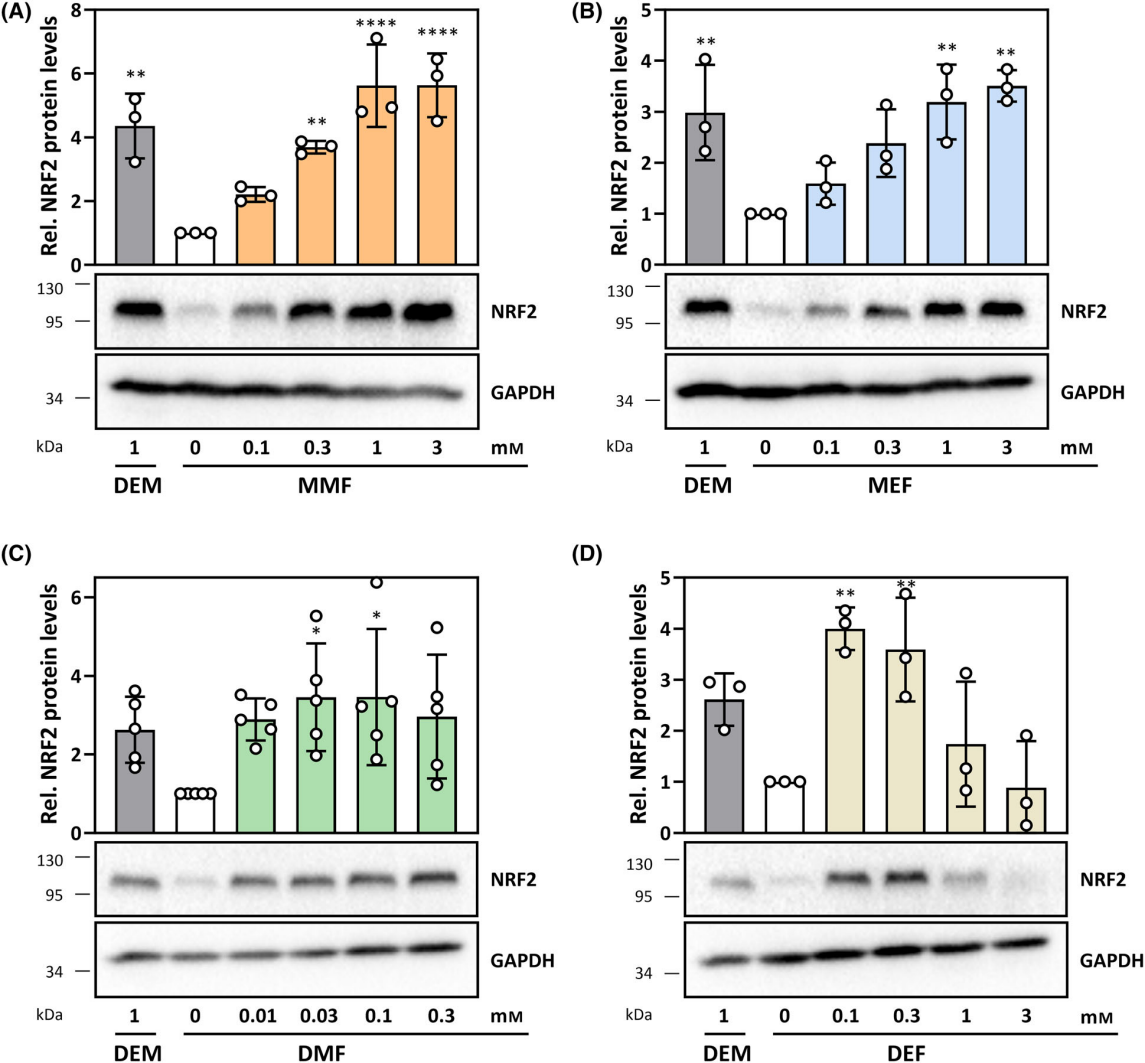
NRF2 protein levels in HepG2 cells exposed to FAEs for 60 min. (A, B) Monomethyl‐ and monoethylfumarate, (C, D) Dimethyl‐ and Diethylfumarate. Diethylmaleate (DEM) was used as positive control in all cases. Blots are representative of at least three independent experiments. Positions of protein markers closest to the detected band are indicated on the left (in kDa). Bar graphs represent densitometric analyses of all experiments, means ± SD. Statistically significant differences from control (white bars) were determined by one‐way ANOVA with Dunnett's *post‐hoc* test. *P < 0.05, **P < 0.01, ****P < 0.0001.

**Fig. 4:**
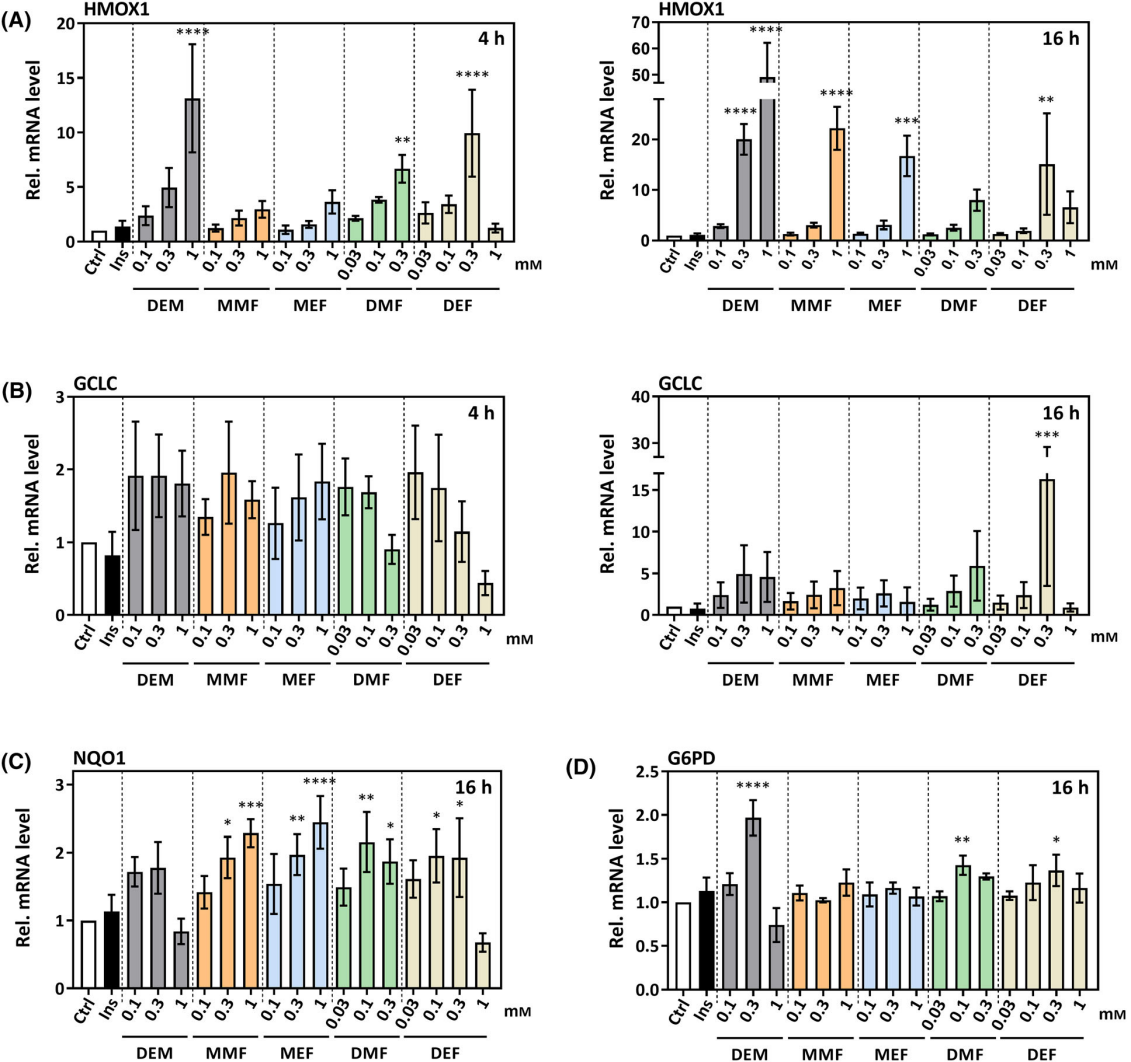
Alterations in mRNA levels of NRF2 target genes in HepG2 cells exposed to FAEs for 4 or 16 h. (A) *HMOX1*, encoding heme oxygenase‐1, (B) *GCLC* (for glutamate‐cysteine ligase, catalytic subunit), (C) *NQO1*, encoding NAD(P)H:quinone oxidoreductase 1, (D) G6PD for glucose 6‐phosphate dehydrogenase. Diethylmaleate (DEM) was used as positive control in all cases. Bars indicate means of three independent experiments ± SD. Statistically significant differences from control (white bars) were determined by one‐way ANOVA with Dunnett's *post‐hoc* test. *P < 0.05, **P < 0.01, ***P < 0.001, ****P < 0.0001.

In contrast to the stark differences between mono‐ and diesters of fumaric acid in acute glutathione depletion and p38^MAPK^ activation, all tested FAEs were capable of significantly stabilizing NRF2 in HepG2 cells (Fig. 3), in turn initiating the upregulation of NRF2 target genes (Fig. 4).

As reported before [18], NRF2 protein levels were very low in non‐stressed (mock‐treated) HepG2 cells (Fig. 3, white bars). An increase in NRF2 levels is indicative of its stabilization due to KEAP1 inactivation and was elicited in a concentration‐dependent manner by MMF and MEF, with induction observed after 60 min of exposure to less than 1 mm of these FAEs (Fig 3A,B). The diesters were even stronger NRF2 activators that required 10‐ (DEF) to 100‐fold (DMF) lower concentrations (Fig 3D, C, respectively), as compared to the monoesters. In the case of DEF, the effect elicited at 0.1 mm even appears to constitute a maximum, as less effect was observed with higher concentrations (Fig. 3D). DEM served as positive control, and induced NRF2 stabilization at the applied concentration of 1 mm (Fig. 3).

Further downstream, the expression of NRF2 target genes was stimulated by FAEs (Fig. 4). Known target genes include those encoding heme oxygenase‐1 (HMOX1), GCLC (glutamate‐cysteine ligase, catalytic subunit), the enzyme catalyzing the rate‐limiting step in GSH biosynthesis, the quinone‐reducing (and thereby antioxidant) enzyme NAD(P)H:quinone oxidoreductase‐1 (NQO1) and glucose 6‐phosphate dehydrogenase (G6PD), the first enzyme in the pentose phosphate pathway, also providing NADPH. Of the tested target genes, *HMOX1* appeared to be the most sensitive toward exposure to FAEs, with highly significant induction observed upon exposure to DEF (like DEM) and DMF already after 4 h. Similarly, a fast and pronounced DEM‐induced upregulation of *HMOX1* mRNA levels in HepG2 cells has been reported before [18]. After 16 h of exposure, signals elicited with diesters still persisted, and strong, highly significant *HMOX1* expression elicited by the monoesters, MMF, MEF, was now observed as well (Fig. 4A). As with NRF2 protein levels (Fig. 3), an apparent maximum active concentration was again observed with DEF at 0.3 mm. *GCLC* gene expression appeared to be slightly elevated over control already at 4 h and at low FAE concentrations, whereas after 16 h DEF was the most strongly stimulating FAE – again with a maximum at 0.3 mm (Fig. 4B). Except for DEF, the observed stimulation of *GCLC* expression remained just below the level of significance. Rather minute, yet (in part highly) significant, effects on gene expression were observed with *NQO1* and (for the diesters) also *G6PD* (Fig 4C, D) after 16 h of exposure, with no more than a two‐fold increase in the respective mRNA levels.

### Fumaric acid diesters stimulate nuclear accumulation of EGFP‐FOXO1

**Fig. 5:**
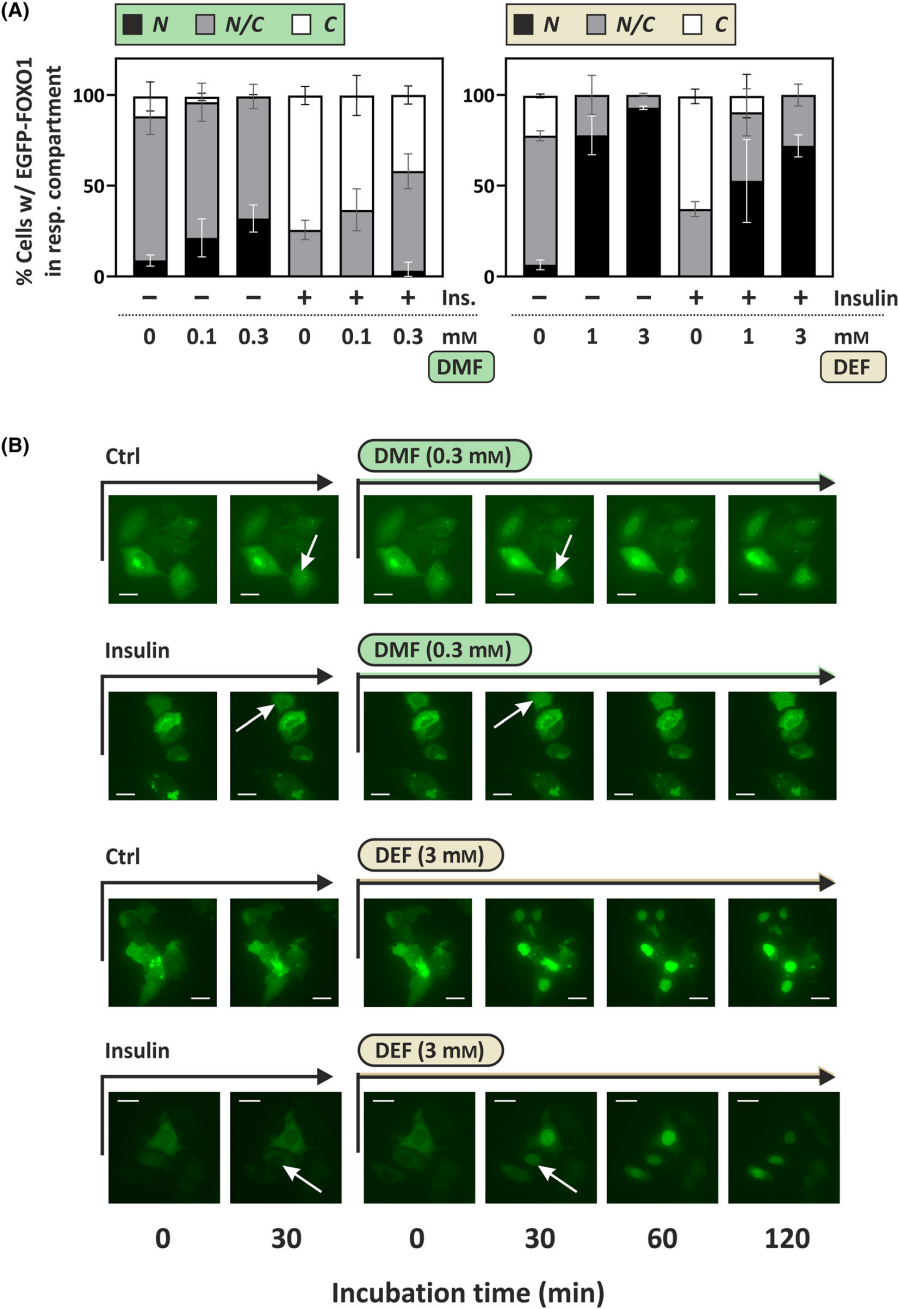
Effects of fumaric acid diesters on subcellular localization of EGFP‐labeled FOXO1. HepG2 cells were transfected with an expression plasmid coding for EGFP‐FOXO1. Prior to exposure of cells to FAEs, cells were pre‐incubated with serum‐free medium for 4–5 h, followed by addition of insulin (Ins., 100 nm) or solvent control for 30 min. Medium was then replaced by serum‐free medium containing FAEs. (A) Cells were exposed to DMF or DEF for 60 min prior to analysis of EGFP‐FOXO1 subcellular localization. Subcellular distribution of EGFP‐FOXO1 in cells was assessed by counting cells with a detectable GFP signal and categorizing them as cells with predominantly nuclear EGFP‐FOXO1 (N), with EGFP‐FOXO1 predominantly in the cytoplasm (C) or cells with EGFP‐FOXO1 equally distributed between the compartments (N/C). The resulting numbers were used to calculate percentages of cells in each category. Data were calculated from three independent experiments. On average, 142 (min: 93, max: 184) cells were categorized per condition in three independent experiments each. Data are given as means ± SD. (B) Cells were prepared as above followed by exposure to insulin (at 100 nm) or control for 30 min and, thereafter, DMF or DEF (or DMSO as control, “0 mm”) for up to 2 h. Scale bars (white) indicate 20 μm.

Shuttling between cytoplasm and nucleus contributes to the regulation of FOXO1 transcriptional activity. Therefore, in order to test for effects of FAEs on FOXO signaling, we first analyzed FOXO1 subcellular localization following exposure of HepG2 cells to FAEs. To that end, we transfected HepG2 cells with a plasmid encoding EGFP‐tagged FOXO1, followed by treatment with insulin and/or FAEs and subsequent detection of the overexpressed EGFP‐FOXO1 by fluorescence microscopy. Treatment of the cells with insulin prior to exposure to FAEs occurred in order to elicit nuclear exclusion of FOXO1, which would then enable us to observe any potential activating effects of FAEs in analogy to DEM, which we had previously identified as a stimulator of FOXO nuclear accumulation [21]. As demonstrated in Fig. 5A,B, insulin treatment (different from control treatment, Ctrl) in fact strongly stimulated nuclear exclusion, as nuclei appear as darker spheres in EGFP‐positive cells after 30 min of insulin treatment.

We then tested for the effects of the fumaric acid diesters, DMF and DEF, with respect to EGFP‐FOXO1 nuclear accumulation. DEF, like its isomer DEM [21], elicited strong nuclear accumulation, even following insulin‐induced nuclear exclusion. DEF, therefore, appears to override the effects of insulin. The effect was strong enough to be detectable also in cells not pre‐treated with insulin (Fig. 5A). The response to DMF exposure was less pronounced, but nevertheless following the same trend: DMF supported nuclear accumulation of EGFP‐FOXO1 (Fig 5A,B). The relatively weak DMF effect may be attributed to the lower achievable concentrations that were used. On the other hand, these lower concentrations also elicited effects on parameters such as GSH depletion (Fig. 1C) or p38^MAPK^ activation (Fig 2C,D) and NRF2 stabilization (Fig 3C,D) that are similar in extent to those of higher DEF concentrations. Another reason for a difference in effect intensity of FAEs may be the reactivity toward protein substrates that are involved in regulating upstream signaling, resulting in FOXO1 modulation. Therefore, we next tested for signaling events up‐ and downstream of FOXO1 nuclear accumulation.

### FAE‐induced nuclear accumulation of FOXO1 is independent of modulation of AKT/FOXO1 signaling

In the insulin signaling cascade, the Ser/Thr kinase AKT is immediately upstream of FOXO1. AKT activation (through phosphorylation at Thr‐308 and Ser‐473) is elicited by exposure of cells to growth factors, such as insulin [23]. AKT, once activated, phosphorylates FOXO proteins, resulting in their inactivation and (in the case of isoforms FOXO1, FOXO3 and FOXO4) nuclear exclusion [9].

**Fig. 6:**
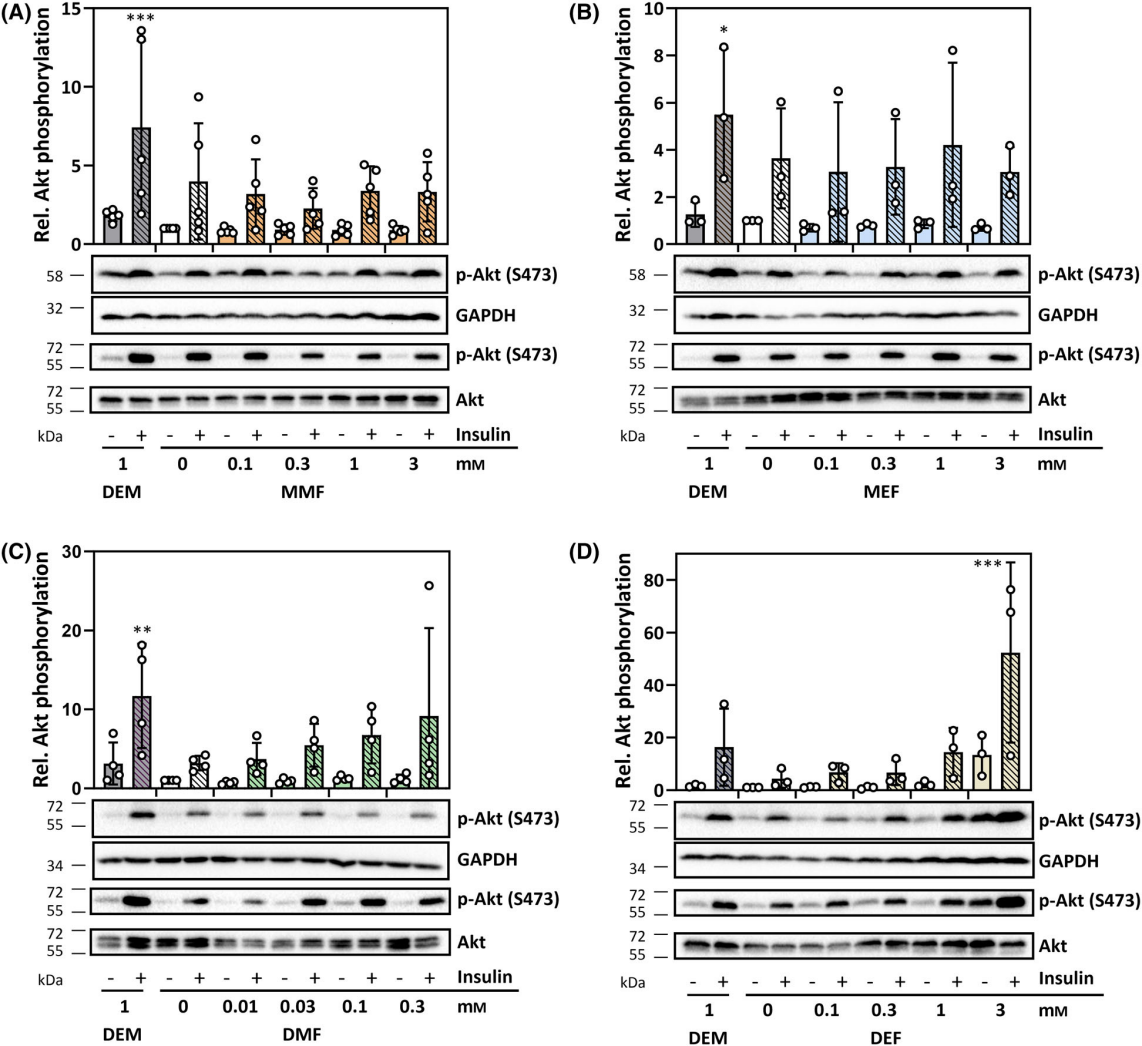
Phosphorylation of AKT in HepG2 cells exposed to FAEs for 60 min. (A, B) Monomethyl‐ and monoethylfumarate, (C, D) dimethyl‐ and diethylfumarate. HepG2 cells were pre‐incubated with serum‐free medium for 16 h, followed by exchange of medium for fresh serum‐free medium containing insulin (100 nm) or a buffer control for 30 min. Insulin was used as a positive control for AKT activation. Cells were washed with serum‐free medium and exposed to fresh serum‐free medium containing monomethyl‐ or monoethylfumarate, dimethyl‐ or diethylfumarate or DEM at the indicated final concentrations for 60 min. Cells were washed twice with cold PBS, lysed, and tested for AKT activation using an antibody detecting Ser‐473 phosphorylation. Blots are representative of three (MEF, DEF), four (DMF), or five (MMF) independent experiments (biological replicates). SDS/PAGE/western Blot were performed twice (two technical replicates) for all experiments (except MMF). Two different replicates are shown for all experiments (one with GAPDH, the other with AKT detected as loading control). Positions of protein markers closest to the detected band are indicated on the left (in kDa). Bar graphs represent densitometric analyses of all experiments (means ± SD). Statistically significant differences were determined by one‐way ANOVA with Dunnett's *post‐hoc* test. *P < 0.05, **P < 0.01, ***P < 0.001.

**Fig. 7:**
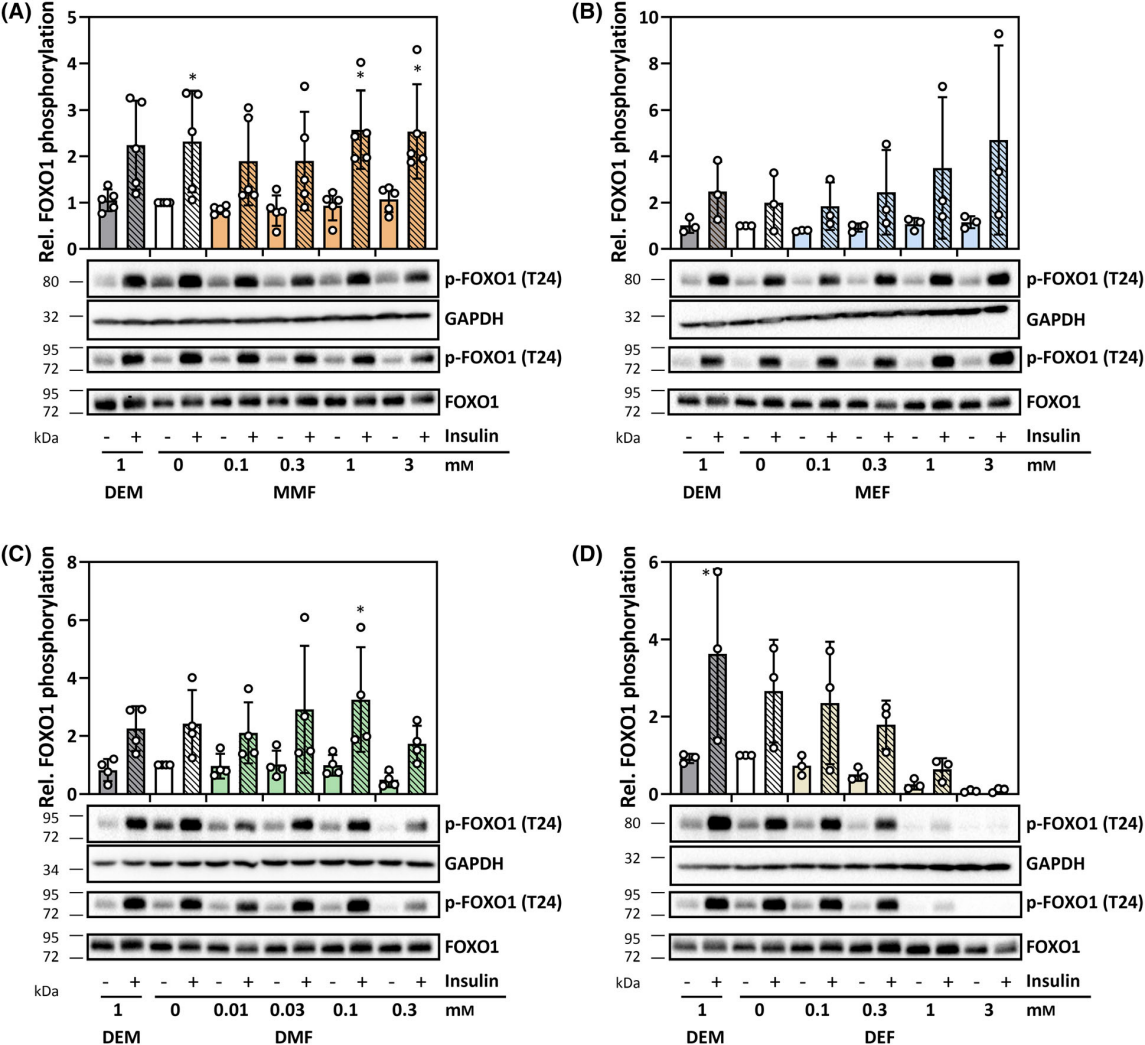
Phosphorylation of FOXO1 in HepG2 cells exposed to FAEs for 60 min. (A, B) Monomethyl‐ and monoethylfumarate, (C, D) dimethyl‐ and diethylfumarate. HepG2 cells were treated as described in the legend to Fig. 6. Insulin was used as a positive control for stimulation of phosphorylation of FOXO1. Following treatments, cells were washed twice with cold PBS, lysed, and tested for FOXO1 inactivation by detecting phosphorylation of a known AKT substrate site, Thr‐24. Phosphorylation of FOXO1 at Thr‐24 was determined by immunoblotting and subsequent densitometric analysis of the blots, with normalization against Ponceau S‐stained protein bands. For each experiment, two different replicates are shown (one with GAPDH, the other with total FOXO1 detected as loading control). Positions of protein markers closest to the detected band are indicated on the left (in kDa). A minimum of three independent experiments was performed for each of the tested compounds; bar graphs represent means ± SD. Statistically significant differences were determined by one‐way ANOVA with Dunnett's *post‐hoc* test. *P < 0.05.

In order to test whether the observed nuclear accumulation elicited by exposure of HepG2 cells to FAEs (Fig. 5) corresponds with AKT inactivation (dephosphorylation) and suppression of AKT‐dependent FOXO1 phosphorylation, we exposed cells to FAEs accordingly, in part following a pre‐stimulation by insulin. Insulin treatment was intended to elevate AKT/FOXO1 phosphorylation levels to allow for better observation of a potential dephosphorylation following FAE exposure. Insulin treatment indeed elicited a strong phosphorylation of AKT (Ser‐473) (Fig. 6), as well as FOXO1 (at the AKT substrate site Thr‐24) (Fig. 7). FAEs, however, did not cause a loss of AKT phosphorylation – neither of basal nor of insulin‐induced phosphorylation (Fig. 6). On the contrary, a slight increase of insulin‐induced AKT activation was observed in cells exposed to the diesters, DMF, DEF and DEM. DEF, at 3 mm, even stimulated AKT phosphorylation in the absence of insulin and enhanced insulin‐induced activation.

No major changes of basal or insulin‐induced FOXO1 phosphorylation at Thr‐24 were observed in cells exposed to FAEs, except for DEF (Fig. 7). DEF treatment dose‐dependently suppressed FOXO1 phosphorylation. This is in sharp contrast to DEF‐induced AKT activation (Fig. 6), which would be expected to result in FOXO phosphorylation. DEF therefore appears to stimulate a process overriding the AKT‐FOXO axis. The mechanism of DEF‐induced FOXO1 dephosphorylation is unclear at this point, but it is in line with FOXO1 nuclear accumulation elicited by DEF (Fig. 5).

### FAEs tend to attenuate rather than stimulate FOXO‐dependent gene expression

**Fig. 8:**
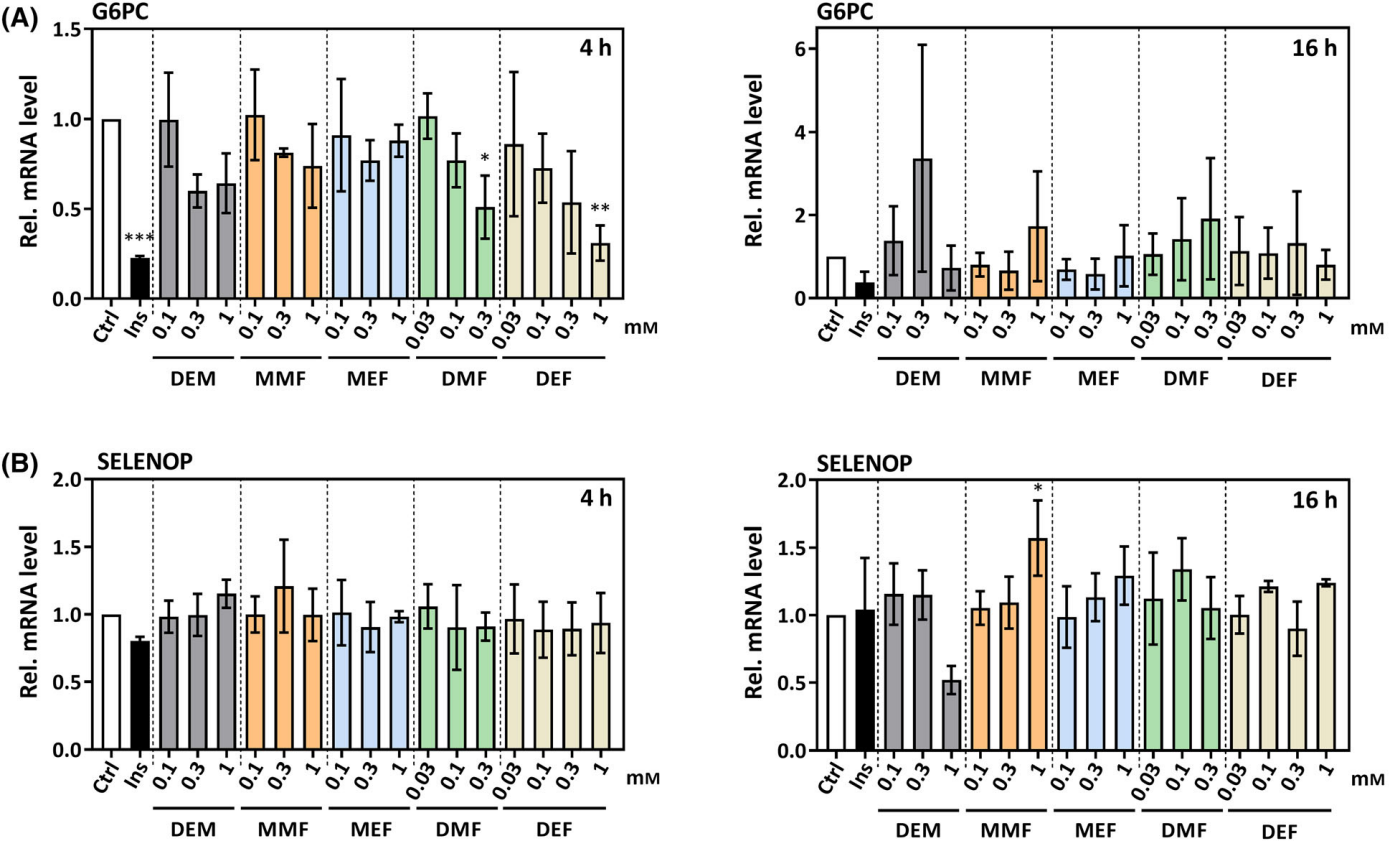
Expression of FOXO target genes in HepG2 cells exposed to FAEs or diethylmaleate (DEM) for 4 or 16 h. (A) *G6PC*, encoding the catalytic subunit of glucose 6‐phosphatase, (B) *SELENOP* (for selenoprotein P). Bars indicate means of three independent experiments ± SD. Statistically significant differences were determined by one‐way ANOVA with Dunnett's *post‐hoc* test. *P < 0.05, **P < 0.01, ***P < 0.001.

In order to test for consequences of FOXO1 nuclear accumulation for gene expression, mRNA levels of two FOXO target genes, *G6PC* [24] and *SELENOP* [25] were analyzed in cells exposed to FAEs for up to 16 h. Insulin was used as positive control, provoking AKT‐induced FOXO inactivation and, consequently, downregulation of FOXO target gene expression. This was most obvious with *G6PC* (Fig. 8A), both at 4 and 16 h of insulin treatment. SELENOP mRNA levels were also downregulated by insulin, but only transiently, at 4 h, and not as strongly as those of *G6PC* (Fig. 8B).

No clear long‐term changes in expression of *G6PC* or *SELENOP* were observed following exposure to FAEs. A transient (i.e., detected at 4 h, but not 16 h) downregulation of *G6PC* expression was observed with the diesters, DMF, DEF, and a similar trend with DEM (Fig. 8A). This is in contrast to expectations, as a DEF‐induced decrease in FOXO phosphorylation and enhanced nuclear accumulation of FOXO would be predicted to result in a stimulation, rather than attenuation, of target gene expression.

Therefore, FAEs may cause FOXO1 nuclear accumulation, but nuclear FOXO1 is not productive in terms of stimulating target gene expression.

## Discussion

All FAEs tested in this study, both mono‐ and diesters, increased NRF2 protein levels and induced NRF2‐dependent signaling in HepG2 human hepatoma cells. Besides hydroxy‐carboxylic acid receptor 2 (HCAR2) and glyceraldehyde‐3‐phosphate dehydrogenase (GAPDH), KEAP1 was listed as one of the key target proteins considered relevant for the mode of action of DMF in a recent edition of an encyclopedia of molecular pharmacology [26]. Considering the reactivity as α,β‐unsaturated carbonyl compounds (see Fig. 1D) that suggest pleiotropic action of FAEs, however, this list is unlikely to be exhaustive. In line with this, FAE target molecules include the major low‐molecular‐mass thiol, glutathione, which is a crucial contributor to cellular antioxidant defense systems as well as to xenobiotic metabolism [27]. Therefore, as alkylating properties are generally accepted as contributing to NRF2 activation by FAEs, we tested for acute depletion of cellular glutathione as a measure of alkylation. No effect was observed with the fumaric acid monoesters MMF and MEF whereas the diesters DMF and DEF (as well as the positive control DEM) caused a glutathione depletion. Despite these differences between fumaric acid mono‐ and diesters, the monoesters were also capable of inducing a stabilization of NRF2, leading to stimulation of NRF2 target gene expression. Induction of expression of the most sensitive target gene, *HMOX1*, was delayed upon exposure to monoesters relative to diesters, suggesting that diesters are more potent stimulators of NRF2 signaling.

Based on *in vitro* [28, 29] and *in vivo* [30] data suggesting hydrolysis of DMF under physiological conditions, it was proposed that the active form of DMF is MMF. MMF reaches the systemic circulation following oral administration of DMF, whereas DMF does not even appear to show up in portal blood (for a comprehensive review of pharmacokinetic data, see ref. [22]). This would imply that DMF is mainly a prodrug. For example, DMF‐induced activation of the G protein‐coupled HCAR2, which is linked to immunomodulatory NF‐κB‐dependent signaling [14], is through MMF: HCAR2 is bound and activated by MMF, but not DMF [31], and was demonstrated to mediate therapeutic effects of DMF in a mouse model of MS [32]. It remains to be elucidated whether, and to what extent, DMF has activities distinct from those of MMF. For example, DMF permeates membranes better than MMF and is much more active as a Michael reagent [33], alkylating cysteines of target proteins, such as KEAP1 [34] and GAPDH [35]. On the other hand, MMF was demonstrated to also target cysteine residues of proteins, including GAPDH [36] (Fig. 1D).

Considering the observed differences in acute glutathione depletion upon exposure to FAEs (Fig. 1C), and the reported capability of monoesters to alkylate protein thiols, it is proposed that both mono‐ and diesters are capable, in principle, of alkylating thiols *in vivo*, but that this is affected by their general differences in avidity to undergo alkylation and by their different membrane permeation properties.

The KEAP1/NRF2 system is a major xenosensor involved in the regulation of cellular antioxidative and stress response and adaptation [2]. Accordingly, NRF2 signaling is widely accepted as a crucial component of therapeutic and beneficial actions of FAEs [15, 22]. Similarly, FOXO transcription factors are involved in a multitude of cellular processes, including the regulation of apoptosis, cell cycle, fuel metabolism, and stress response. Again, there is ample evidence for redox regulation of FOXO signaling, including the role of protein thiols in the signaling cascades affecting FOXO activity (for review, see ref. [4, 9]). A multitude of stimuli were identified that affect both NRF2 and also FOXO (or upstream) signaling, including reactive oxygen species such as H_2_O_2_ [8, 37] or peroxynitrite [38, 39, 40], lipid peroxidation products such as 4‐hydroxynonenal [41, 42], flavonoids [8, 10], transition metal ions and metalloids, such as Cu [6, 43, 44], Zn [45, 46] or As [19, 37, 47].

Why, then, do FAEs not significantly affect FOXO signaling in HepG2 cells, while clearly stimulating NRF2‐dependent gene expression? Several distinctive features of FOXO‐ and KEAP1/NRF2 signaling can be delineated in the context of an exposure to FAEs:

- KEAP1/NRF2 appears to be generally more sensitive to FAEs than the signaling cascade(s) leading to modulation of FOXO activity. While we are not aware of an indication that KEAP1 cysteines are more easily oxidized than sensitive protein thiols in pathways affecting FOXOs, there may be a selectivity with respect to thiol alkylation. In fact, of all KEAP1 cysteines, only specific Cys residues (predominantly Cys151) were identified to be alkylated by fumarates [48], implying that not all Cys thiols are equally prone to be attacked by fumaric acid esters. Importantly, however, there is indication of an additional mode of interaction of FAEs (as demonstrated for DMF and MMF) with KEAP1, as they may also bind to KEAP1 noncovalently, additionally interfering with KEAP1/NRF2 interaction [49]. No such noncovalent interaction of FAEs has been demonstrated with components of FOXO‐modulating pathways.
- Although the same stress‐activated kinases, including ERK^MAPK^, JNK^MAPK^, and p38^MAPK^ (see Fig. 2) as well as AKT (Fig. 6) modulate both FOXO and NRF2 activity, the outcome of an activation of these kinases appears to be mostly stimulating with respect to NRF2, but highly variable with respect to FOXO activity. For example, stress‐induced activation of ERK^MAPK^, JNK^MAPK^ and p38^MAPK^ may cause their cooperative stimulation of NRF2 [50], whereas their effect on FOXO activity varies with FOXO isoform, site phosphorylated and cell type [9]. Similarly, AKT activation causes phosphorylation of FOXOs, resulting in their nuclear exclusion and inactivation. NRF2 activity, however, is stimulated: AKT phosphorylates and thereby inactivates glycogen synthase kinase 3 (GSK3), thereby attenuating NRF2 phosphorylation by GSK3, which results in an attenuation of NRF2 degradation (and thereby its activation) [51]. Hence, AKT activation may differentially affect transcriptional regulation by FOXO (which is inactivated) and NRF2 (which is activated). In the present study, however, such an effect of AKT is unlikely, as no basal AKT activation was observed with FAEs – except for the highest concentration of DEF (Fig. 6).
- In addition to KEAP1 alkylation and noncovalent binding to KEAP1, impairment of a repressor of NRF2 signaling, BACH1, was proposed to contribute to FAE‐induced NRF2 activation: both DMF and MMF were shown in a cell culture model (human M17 neuroblastoma cells) to elicit nuclear export of BACH1, with DMF effects being more pronounced [52]. DMF was also demonstrated to non‐specifically alkylate overexpressed BACH1 in cultured Cos‐7 cells [53]. It is unclear whether such alkylation contributes to BACH1 nuclear exclusion. In contrast to these reports for BACH1, nuclear export of FOXO1 was previously hypothesized to be impaired by the *cis*‐isomer of DEF, DEM [21]. Based on reports that DEM interferes with nuclear export mediated by exportin CRM1 [54], which is a known mediator of FOXO1 nuclear exclusion [21], one would have to hypothesize that nuclear export impairment by FAEs is selective rather than general.

In summary, despite the similarities in NRF2 and FOXO signaling with respect to their being modulated by similar stressful stimuli and with respect to their stimulating the expression of genes encoding antioxidant enzymes, only NRF2 signaling was activated by FAEs in HepG2 cells. Both mono‐ and diesters were capable of eliciting a NRF2 response, thus activating NRF2‐dependent expression of potentially hepatoprotective factors. FOXO1, however, despite nuclear accumulation upon exposure of cells to FAEs, was not activated under conditions that lead to NRF2 activation.

## Conflict of interest

The authors declare no conflict of interest.

## Author contributions

Conceptualization, LOK; investigation, KE, NK; data curation, KE, NK; writing—original draft preparation, LOK; writing—review and editing, all authors; supervision, LOK.

## Data Availability

The data presented in this study are available upon reasonable request from the corresponding author.
